# Characterization of the complete chloroplast genome of a well-known Chinese medicinal herb, *Scrophularia ningpoensis*

**DOI:** 10.1080/23802359.2019.1705926

**Published:** 2020-01-13

**Authors:** Hong-Lian Ai, Chun-Lun Qin, Ke Ye, Zheng-Hui Li

**Affiliations:** School of Pharmaceutical Sciences, South-Central University for Nationalities, Wuhan, China

**Keywords:** Chloroplast genome, medicinal herb, *Scrophularia ningpoensis*, phylogenetic analysis

## Abstract

*Scrophularia ningpoensis* has been used as a famous traditional medicinal herb in Asian countries to treat jaundice, dysentery, and the pain of rheumatism. In this paper, the complete chloroplast (cp) genome sequence of *S. ningpoensis* was reported and characterized. The cp genome is 153,175 bp in length, composed of a pair of 25,490 bp inverted repeat (IR) regions separated by a large single copy (LSC) region of 84,257 bp and a small single copy (SSC) region of 17,938 bp. There were 130 predicted genes (85 protein-coding genes, 37 tRNA genes, and 8 rRNA genes) in the genome, and the overall GC content of the genome is 38%. Phylogenetic analysis based on the cp genome data showed that *S. ningpoensis* was sister to *S. buergeriana.*

*Scrophularia ningpoensis* Hemsl. (Scrophulariaceae), a well-known traditional Chinese medicine herb, has been used for centuries in many Asian countries for the promotion of health and longevity (Yu et al. 2017). In clinical practice, it is commonly used to treat pharyngalgia, arthritis, rheumatism, constipation, tussis, conjunctival congestion and various inflammation diseases, and it is especially effective for the treatment of throat and vocal cord (Zhu et al. 2013; Ma et al. 2019; Su et al. 2019). Here, we reported the complete chloroplast (cp) genome sepuence of *S*. *ningpoensis*, and revealed its phylogenetic relationships with other species in the *Scrophularia*.

Fresh leave materials of *S*. *ningpoensis* were collected from Longping town of Jianshi county, Hubei, China (N30°48′24″, E110°1′47″, 1,750 m). Meanwhile, the voucher specimen (HSN12443) were deposited in the Herbarium of South-Central University for Nationalities (HSN). The total genomic DNA of *S*. *ningpoensis* was extracted and sequenced on Illumina HiSeq 4000 Platform at the Beijing Novogene Bioinformatics Technology Co., Ltd (Nanjing, China). About 2 Gb raw data were used to *de novo* assemble the complete cp genome using SPAdes (Bankevich et al. 2012). The complete genome sequence was annotated using Dual Organellar Genome Annotator (DOGMA) (Wyman et al. 2004) with manual adjustments. The sequence of cp genome was deposited in GenBank (accession numbers MN734369).

The complete cp genome of *S*. *ningpoensis* represents a typical quadripartite circular molecule with 153,175 bp in size. This genome is composed by a small single copy (SSC) region of 17,938 bp, a large single copy (LSC) region of 84,257 bp and a pair of inverted repeat (IR) regions of 25,490 bp. The GC content of this genome is 38%. There were a total of 130 genes including 85 protein-coding genes, 37 tRNA genes, and 8 rRNA genes in the genome. Most of the genes occurred in a single copy, while four rRNA genes (i.e. 4.5S, 5S, 16S, and 23S rRNA), seven tRNA genes (i.e. *trnA-UGC*, *trnI-CAU*, *trnI-GAU*, *trnL-CAA*, *trnN-GUU*, *trnR-ACG*, and *trnV-GAC*) and six protein-coding genes (i.e. *rpl2*, *rpl23*, *ycf2*, *ndhB*, *rps7*, and *rps12*) occurred in double. Among the 130 genes, twenty of them had one intron, and three had two introns (*clpP*, *rps12* and *ycf3*).

To identify the phylogenetic position of *S*. *ningpoensis*, phylogenetic analysis was conducted based on the complete cp genome of this species and other six species belonging to Scrophulariaceae. The sequences were aligned with MAFFT (Katoh and Standley 2013). The maximum-likelihood (ML) and Bayesian inference (BI) phylogenetic trees were reconstructed using RAxML (Stamatakis 2014) and MrBayes (Ronquist et al. 2012). The ML and BI analyses generated the same tree topology (Figure 1). As shown in the phylogenetic tree (Figure 1), *S*. *ningpoensis* was closely related to *S. buergeriana* with 100% bootstrap and 1.0 posterior probability support, respectively. Our findings will provide a foundation for further investigation of cp genome evolution in *Scrophularia*, and also could be further applied for evolutionary and phylogenetic studies of *Scrophularia*.

**Figure 1.**
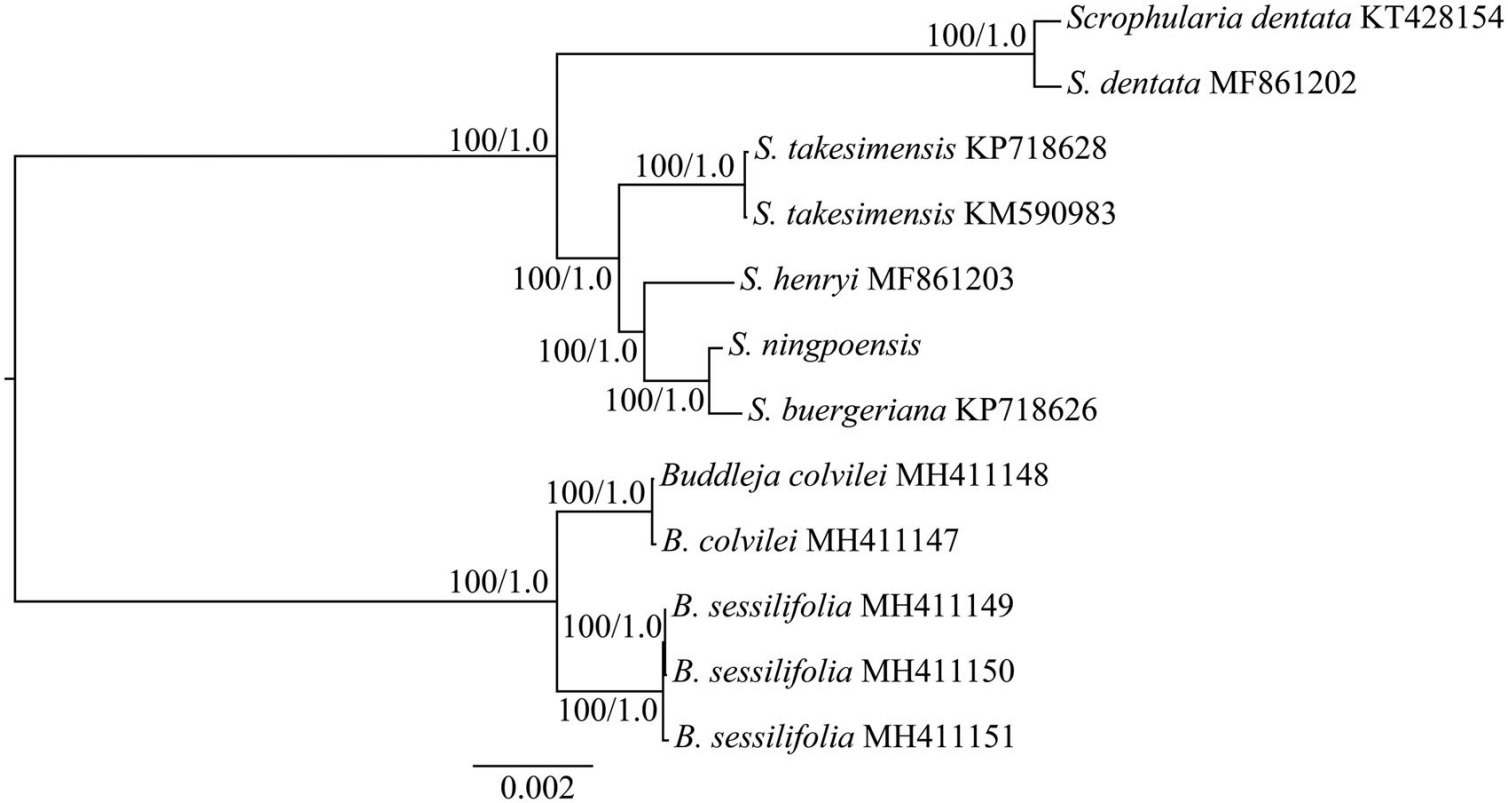
The maximum likelihood (ML) tree of Scrophulariaceae inferred from the complete chloroplast genome sequences. Numbers at nodes correspond to ML bootstrap percentages (1,000 replicates) and Bayesian inference (BI) posterior probabilities.
